# STAT3 Decoy Oligodeoxynucleotides-Loaded Solid Lipid Nanoparticles Induce Cell Death and Inhibit Invasion in Ovarian Cancer Cells

**DOI:** 10.1371/journal.pone.0124924

**Published:** 2015-04-29

**Authors:** Yanhui Ma, Xiaolei Zhang, Xiaoxuan Xu, Liang Shen, Yao Yao, Ziyan Yang, Peishu Liu

**Affiliations:** 1 Department of Obstetrics and Gynecology, Qilu Hospital of Shandong University, Jinan, Shandong, China; 2 Department of Obstetrics and Gynecology, Provincial Hospital Affiliated with Shandong University, Jinan, Shandong, China; 3 Department of Pharmaceutics, School of Pharmaceutical Sciences, Shandong University, Jinan, Shandong, China; Universidad de Castilla-La Mancha, SPAIN

## Abstract

Recent advances in the synthesis of multi-functional nanoparticles have opened up tremendous opportunities for the targeted delivery of genes of interest. Cationic solid lipid nanoparticles (SLN) can efficiently bind nucleic acid molecules and transfect genes *in vitro*. Few reports have combined SLN with therapy using decoy oligodeoxynucleotides (ODN). In the present study, we prepared SLN to encapsulate STAT3 decoy ODN; then, the properties and *in vitro* behavior of SLN-STAT3 decoy ODN complexes were investigated. SLN-STAT3 decoy ODN complexes were efficiently taken up by human ovarian cancer cells and significantly suppressed cell growth. Blockage of the STAT3 pathway by SLN-STAT3 decoy ODN complexes resulted in an evident induction of cell death, including apoptotic and autophagic death. The mechanism involved the increased expression of cleaved caspase 3, Bax, Beclin-1 and LC3-II and reduced expression of Bcl-2, pro-caspase 3, Survivin, p-Akt and p-mTOR. In addition, SLN-STAT3 decoy ODN complexes inhibited cell invasion by up-regulating E-cadherin expression and down-regulating Snail and MMP-9 expression. These findings confirmed that SLN as STAT3 decoy ODN carriers can induce cell death and inhibit invasion of ovarian cancer cells. We propose that SLN represent a potential approach for targeted gene delivery in cancer therapy.

## Introduction

Ovarian cancer is the most lethal female gynecologic malignancy, causing approximately 15,500 deaths annually in the United States [1]. Recurrence and subsequent resistance to chemotherapy result in low cure rates and a high mortality rate [2]. The five-year survival rate remains low. Therefore, there is an urgent need for alternative, effective regimens.

Gene therapy has been reported to possess tremendous potential in biomedical applications, including with respect to neoplastic, genetic and infectious diseases [3–6]. The Janus kinase/signal transducer and activator of transcription (JAK/STAT) signaling pathway plays an important role in transferring signals from the plasma membrane to the nucleus. STAT3 is a crucial member of the STAT family and has been recognized as an oncogene [7]. Constitutive STAT3 activation has been found in ovarian cancer cell lines and clinical specimens [8–10]. Recent evidence has shown that the constitutive activation of STAT3 is closely related to cell growth, differentiation, survival and metastasis [11–13]. STAT3 may be a potential target of cancer therapy [14]. The strategy of using decoy oligodeoxynucleotides (ODN) is a promising approach to inhibiting the STAT3 pathway. STAT3 decoy ODN are 15-mer double-stranded oligonucleotides that mimic DNA binding consensus sequences, acting primarily by competitive binding to activated STAT3 dimers [15]. It has been reported that the STAT3 decoy ODN can suppress the growth of human glioma cells, hepatocellular carcinoma cells and colorectal carcinoma cells via blocking nuclear transfer [16–18]. STAT3 decoy ODN provide hope for decoy ODN-based cancer therapy.

However, naked ODN are rapidly degraded by nucleases with poor biomembrane permeability; therefore, a crucial issue is the development of efficient and safe gene carriers for delivering ODN into target cells and ensuring their stability to exert their biological effect [19–22]. Cationic solid lipid nanoparticles (SLN) represent a new generation of delivery carriers among lipid dispersions since more than two decades. SLN are composed of physiological lipids dispersed in an aqueous surfactant solution. The cationic nanostructure can bind anionic nucleic acids through ionic interactions; and protect the encapsulated nucleic acid molecules from degradation by nucleases to regulate their release [23]. In addition, the availability of steam sterilization, lyophilization, and mass production make SLN quite appropriate as a gene delivery system [24]. Our group has successfully prepared SLN using a solvent displacement technique and performed systemic analysis of SLN. Results show that SLN have a strong DNA binding capacity, low cytotoxicity and high gene transfection efficiency [25].

Due to the advantages of decoy ODN and SLN, herein, we incorporated STAT3 decoy ODN into SLN to form SLN-STAT3 decoy ODN complexes and detected their characteristics as well as their uptake behavior in the human ovarian cancer cell lines SKOV3 and A2780. After the complexes were internalized in cells, STAT3 decoy ODN were released from the nanostructures.

Apoptosis and autophagy are types of programmed cell death that play a pivotal role in cancer therapy. Local invasion or distant metastasis are common in ovarian cancer and are associated with poor prognosis. Therefore, we studied the effects of SLN-STAT3 decoy ODN complexes on cell death (including apoptosis and autophagy) and invasion. Our data indicate that the complexes block the STAT3 pathway and exert a biological effect. These results may provide information regarding the enormous potential of gene delivery in the targeted therapy of ovarian cancers.

## Materials and Methods

### SLN preparation and characterization

The solvent diffusion method was used to prepare SLN under optimal conditions using cetyltrimethylammonium bromide (CTAB, Amersco, USA) as a cationic surfactant as reported previously [25]. Briefly, glyceryl monostearate (20 mg, Shanghai Chemical Reagent Co., Ltd. China) and soya lecithin (15 mg, Shanghai Pujiang Phospholipids Co., Ltd. China) were added to 2 ml of acetone followed by ultrasonication to form the organic phase. CTAB (15 mg) was dissolved in 20ml of deionized water to form the aqueous phase. The organic phase was injected into the aqueous phase at 12 ml/h under magnetic stirring at 400 rpm (ETS-D4 stirrer, IKA, Germany) at room temperature. The solution was stirred for 12 hours to evaporate the organic solvents. The resultant suspensions were separated by centrifugation at 15,000 rpm. The obtained nanoparticles were washed three times, redissolved in deionized water, and filtered through a membrane with a 0.45-μm pore size, and the PH was adjusted to 7.2–7.4. The SLN suspensions were stored at 4°C. Transmission electronic microscopy (TEM) was performed to observe the morphology of the SLN (JEM-1200EX, Japan). The particle size and zeta potential of the SLN were analyzed by photon correlation spectroscopy using a Zetasizer 3000 (Malvern Instruments, Malvern, England).

### STAT3 decoy and scrambled ODN

All ODN were synthesized by Sangon Biotech (Shanghai, China), as previously reported, with the following sequences: STAT3 decoy ODN, 5’-CATTTCCCGTAAATC-3’, 3’-GTAAAGGGCATTTAG-5’; STAT3 scrambled ODN, 5’-CATCTTGCCAATATC-3’, 3’-GTAGAACGGTTATAG-5’ [26].

### Preparation of SLN-STAT3 decoy ODN complexes and gel retardation assays

The SLN and STAT3 decoy ODN solution were mixed under gentle vortexing for 20 seconds and incubated for 20 minutes at room temperature. Samples with different SLN/ODN ratios were analyzed by electrophoresis on 2% agarose gel in Tris acetate-EDTA (TAE) buffer at 90 V for 20 minutes. Images were recorded using a UV transilluminator and a digital imaging system (IS-2200, Alpha Innotech, USA) [27].

### Cell culture

Human ovarian cancer cell lines A2780 and SKOV3 were purchased from the Cell Bank of the Chinese Academy of Sciences. A2780 cells were cultured in RPMI 1640 medium (HyClone, Beijing, China) supplemented with 10% fetal bovine serum (FBS; Haoyang Biological manufacture Co., Ltd, Tianjin, China) and 1% penicillin-streptomycin (Invitrogen, USA). SKOV3 cells were cultured in McCoy’s 5A medium (Sigma-Aldrich, USA) containing 10% FBS and 1% penicillin-streptomycin. All cells were cultured in a humidified atmosphere at 37°C and 5% CO_2_.

### Cell Viability Assay

Cells were seeded in 96-well plates and transfected with the SLN-STAT3 decoy ODN complexes at a series of concentrations of ODN (0–50 nmol/L) the next day. After 24, 48 and 72 hours, cell viability was determined using a 3-(4,5-dimethylthiazol-2-yl)-2,5 diphenyltetrazolium bromide (MTT; Sigma) assay.

### Cellular uptake of SLN-decoy ODN complexes

Cells were seeded in 6-well plates. When cells were approximately 50–60% confluent, the complete medium was replaced with Opti-MEM (Gibco, USA) containing SLN-decoy ODN complexes. The culture medium was replaced by complete medium after 6 hours of incubation at 37°C in a 5% CO_2_ incubator. The decoy ODN used for uptake efficiency assay were labeled with fluorescein isothiocyanate (FITC). After 24 or 48 hours of treatment with SLN-decoy ODN complexes, the cellular fluorescence was visualized using an inverted fluorescence microscope (Olympus, Japan). Then, cells were harvested by trypsin, washed with phosphate buffered saline (PBS) and determined by flow cytometry (FCM; BD, San Jose, CA, USA).

### Apoptosis Assay

After 48 hours of transfection, cell apoptosis was measured using an annexin V-FITC/propidium iodide (PI) double-staining assay (the Apoptosis Detection Kit, BestBio, Shanghai, China) with flow cytometry.

### Detection of acidic vesicular organelles (AVOs)

At 48 hours after transfection, cells were fixed in 2.5% glutaraldehyde in 0.1 M cacodylate buffer and then post-fixed in 1% osmium tetroxide with 0.1% potassium ferricyanide. The samples were dehydrated through an ethanol gradient (30–90%) and embedded in Epon. The sample blocks were cut into ultra-thin sections and stained with 2% uranyl acetate. Samples were viewed by TEM. After 48 hours of transfection, cells were stained with acridine orange (AO; 1 μg/ml in PBS) for 15 minutes at 37°C. Samples were observed under an inverted fluorescence microscope.

### Migration and invasion assay

Cells were seeded in 6-well plates and transfected the next day. When the confluence reached approximately 90%, the cells were artificially scratched with a pipette tip and cultured in medium containing 1% FBS. At 0, 24 and 48 hours, images of the scratch wounds were captured. Cell migration was measured as the new scratched width relative to the original scratched width. For the invasion assay, the polycarbonate membrane of a 24-well transwell chamber (Corning Costar, USA) was uniformly coated with 20 μL of Matrigel (BD Biosciences, USA; 1:5 dilution) as the intervening membrane. At 48 hours after transfection, 5 × 10^4^ cells suspended in 200 μL of serum-free medium were seeded in the upper chamber, and 600 μL of complete medium was added to the lower chamber. After incubation for 24 hours at 37°C, the cells that had penetrated to the bottom of the membrane were fixed, stained with crystal violet and counted under a microscope in five random fields.

### Western blot analysis

Equal amounts of the protein samples were separated by 10–12% SDS-PAGE. Blots were incubated with primary antibodies against STAT3, p-STAT3 (Ser727, Tyr705), Bcl-2, Bax, caspase 3, mTOR, p-mTOR, Akt, p-Akt, Beclin-1, LC3A, LC3B, E-cadherin, Snail, MMP-9 (Cell Signaling Technology, USA) and Survivin (R&D Systems Inc.). Horseradish peroxidase-labeled anti-rabbit antibody was used as a secondary antibody (Beijing Zhong Shan Biotech Co., Ltd. Beijing, China). Blots were visualized using enhanced chemiluminescence (ECL; Millipore). The band of β-actin (Beijing Zhong Shan Biotech Co., Ltd. Beijing, China) was served as a loading control. Band density was quantified by Image J software.

### Statistical analyses

GraphPad Prism 5.01 (GraphPad software, USA) was used to perform statistical analyses. All experiments were performed at least three times. The results are expressed as means ± standard deviation (SD). *P* values < 0.05 were considered statistically significant.

## Results

### Preparation and characterization of SLN and SLN-STAT3 decoy ODN complexes

**Fig 1:**
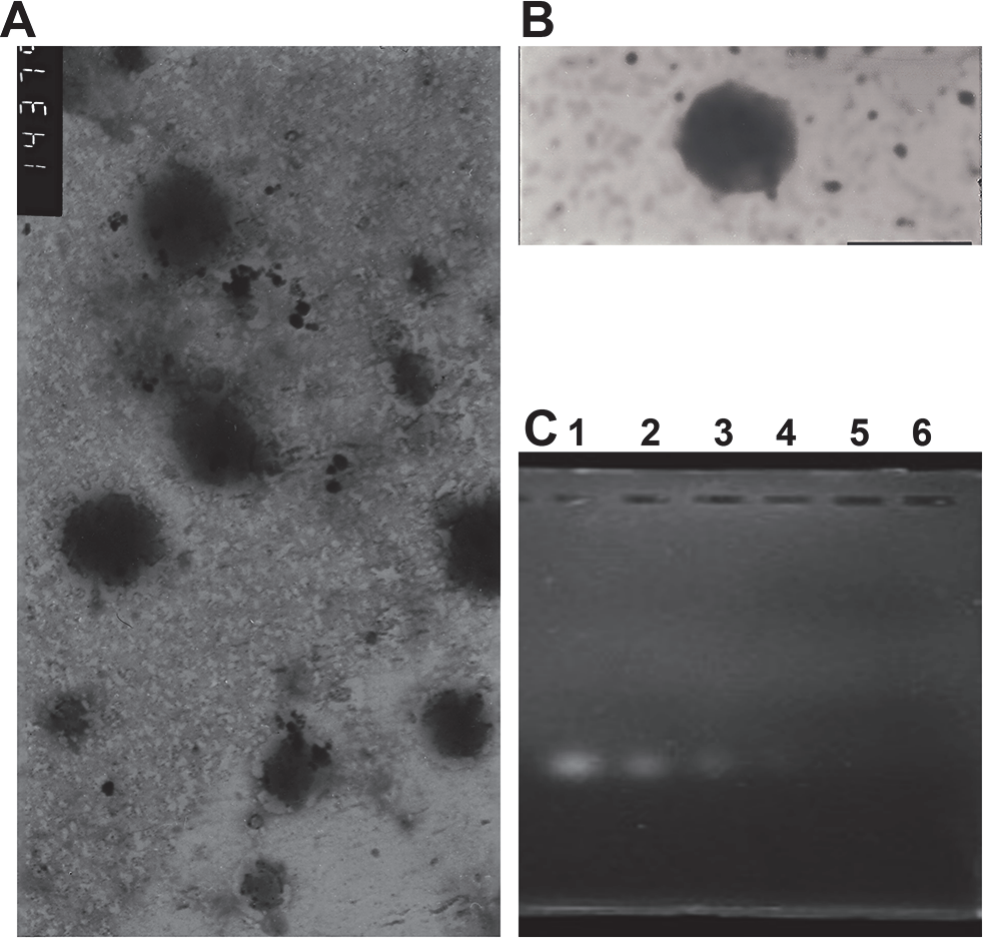
Characterization of SLN and SLN-STAT3 decoy ODN complexes. (A) TEM image of bare SLN (scale bar, 100 nm). (B) TEM image of SLN–STAT3 decoy ODN complexes (scale bar, 100 nm). (C) Gel retardation assay of SLN-decoy ODN complexes. A total of 0.5 μg of decoy ODN per hole was mixed with SLN. The weight ratios of SLN to ODN from left to right are as follows, lane 1: naked ODN control; lane 2: SLN:ODN = 5:1; lane 3: SLN:ODN = 10:1; lane 4: SLN:OND = 20:1; lane 5: SLN:ODN = 50:1; lane 6: SLN:ODN = 100:1.

SLN were successfully synthetized in our study as previously described. The mean particle size of the SLN was 67.53 ± 7.74 nm, with a polydispersity index of 0.25 ± 0.04. The zeta potential was 44.18 ± 3.12 mV. Morphological observation by TEM showed that both the SLN and SLN-STAT3 decoy ODN complexes were spherical or ellipsoidal (Fig 1A and 1B). The formation of SLN-STAT3 decoy ODN complexes was evaluated by agarose gel electrophoresis. SLN and STAT3 decoy ODN were mixed at weight ratios (w/w) of 5:1, 10:1, 20:1, 50:1, and 100:1. SLN completely condensed the ODN at a weight ratio 20:1 (Fig 1C). Thus, the optimal ratio for preparing the complexes was 20:1, which was used in the following experiments. Compared with the SLN, the SLN-STAT3 decoy ODN complexes were larger, and exhibited a lower zeta potential because of the combination of the decoy ODN. The mean particle size of the complexes was 101.30 ± 11.89 nm with a polydispersity index of 0.24 ± 0.03. The value of the zeta potential was 20.03 ± 0.93 mV.

### Cell Viability

**Fig 2:**
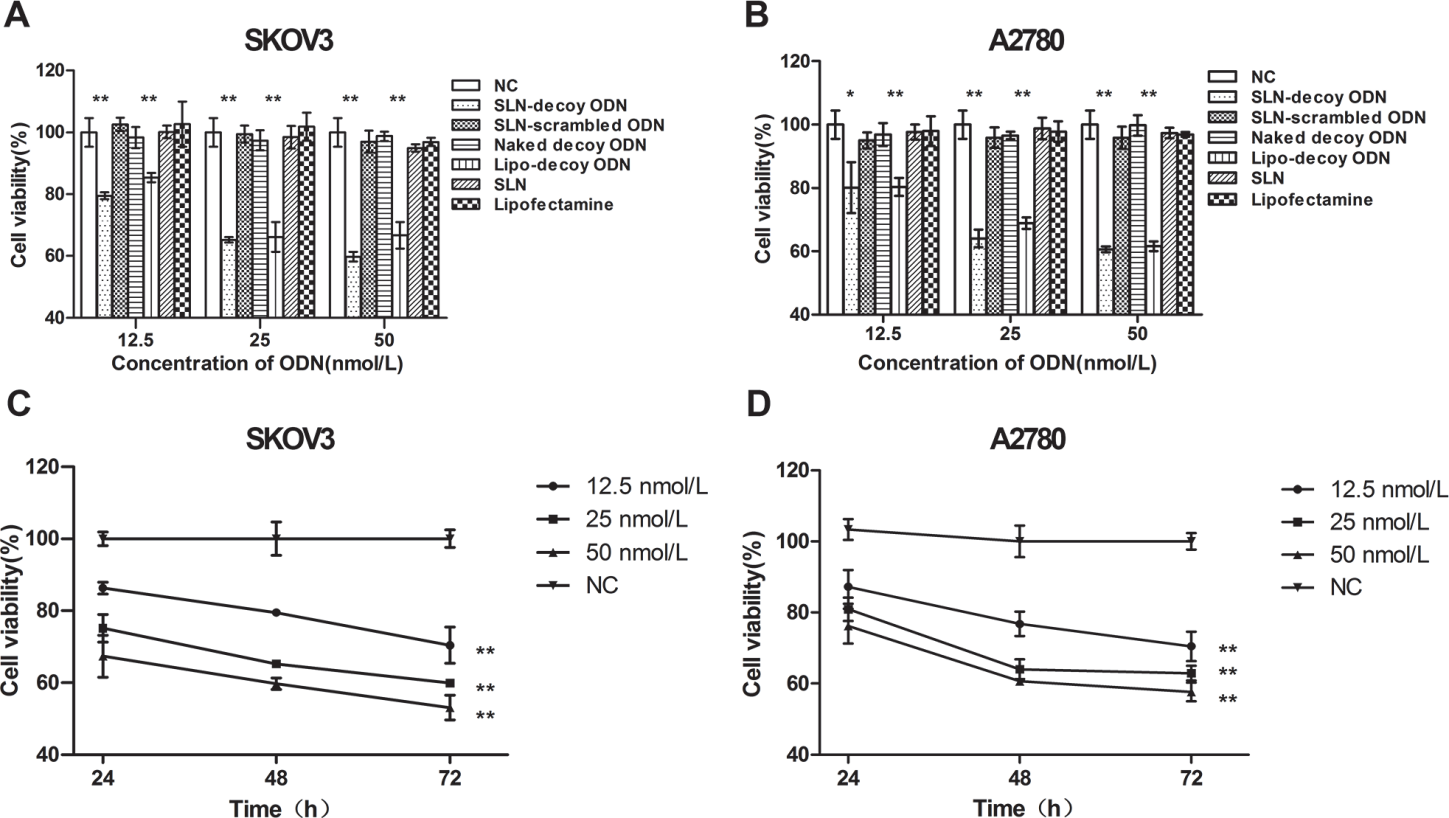
Cell viability of ODN formulations by MTT assay. SKOV3 (A) and A2780 (B) cells were treated with negative agents (negative control, NC), SLN-decoy ODN complexes, SLN-scrambled ODN complexes, naked ODN, Lipofectamine 2000-decoy ODN complexes (Lipo-decoy ODN), SLN and Lipofectamine 2000 at different concentrations (12.5, 25 and 50 nmol/L). At 48 hours after treatment, an MTT assay was performed. SKOV3 (C) and A2780 (D) cells were transfected with serial concentrations of SLN-decoy ODN complexes (12.5, 25 and 50 nmol/L), and cell viability was assessed at 24, 48 and 72 hours. The data are expressed as the means ± SD of three independent experiments, * *P* < 0.05, ** *P* < 0.01 compared with NC.

Our previous study showed that Lipofectamine 2000-STAT3 decoy ODN (Lipo-decoy ODN) can suppress the growth of SKOV3 and OVCAR3 cells. To explore whether SLN-decoy ODN have a similar effect on tumor suppression as Lipo-decoy ODN, SKOV3 and A2780 cells were treated with different concentrations of SLN-decoy ODN and Lipo-decoy ODN. STAT3 scrambled ODN were used to test the sequence specificity of the decoy ODN. Negative control (NC), naked decoy ODN, SLN, and Lipofectamine 2000 groups were used as controls. Cell growth was notably suppressed by the SLN-decoy ODN and Lipo-decoy ODN (*P* < 0.05), although the SLN did not induce a marked growth suppression effect compared with the NC or Lipo groups at each investigated concentration at 48 hours after treatment. The growth suppression of the SLN-decoy ODN showed no significant difference from that of the Lipo-decoy ODN at each dose after treatment for 48 hours. SLN-scrambled ODN complexes and naked decoy ODN indicated no effects on cell proliferation compared with the NC group at each concentration (Fig 2A and 2B; *P* > 0.05). SLN-decoy ODN complexes inhibited cell growth in a time- and dose-dependent manner (Fig 2C and 2D). Considering the cytotoxicity of CTAB, we chose a concentration of 25 nmol/L for the following experiments. These data suggested that both the SLN-decoy ODN and the Lipo-decoy ODN could clearly suppress the growth of ovarian cancer cells. The data also suggested that the suppression effect of SLN-decoy ODN was not due to the nanoparticle carrier because SLN did not show any suppression effect *in vitro*.

### Cellular Uptake of SLN-decoy ODN complexes

**Fig 3:**
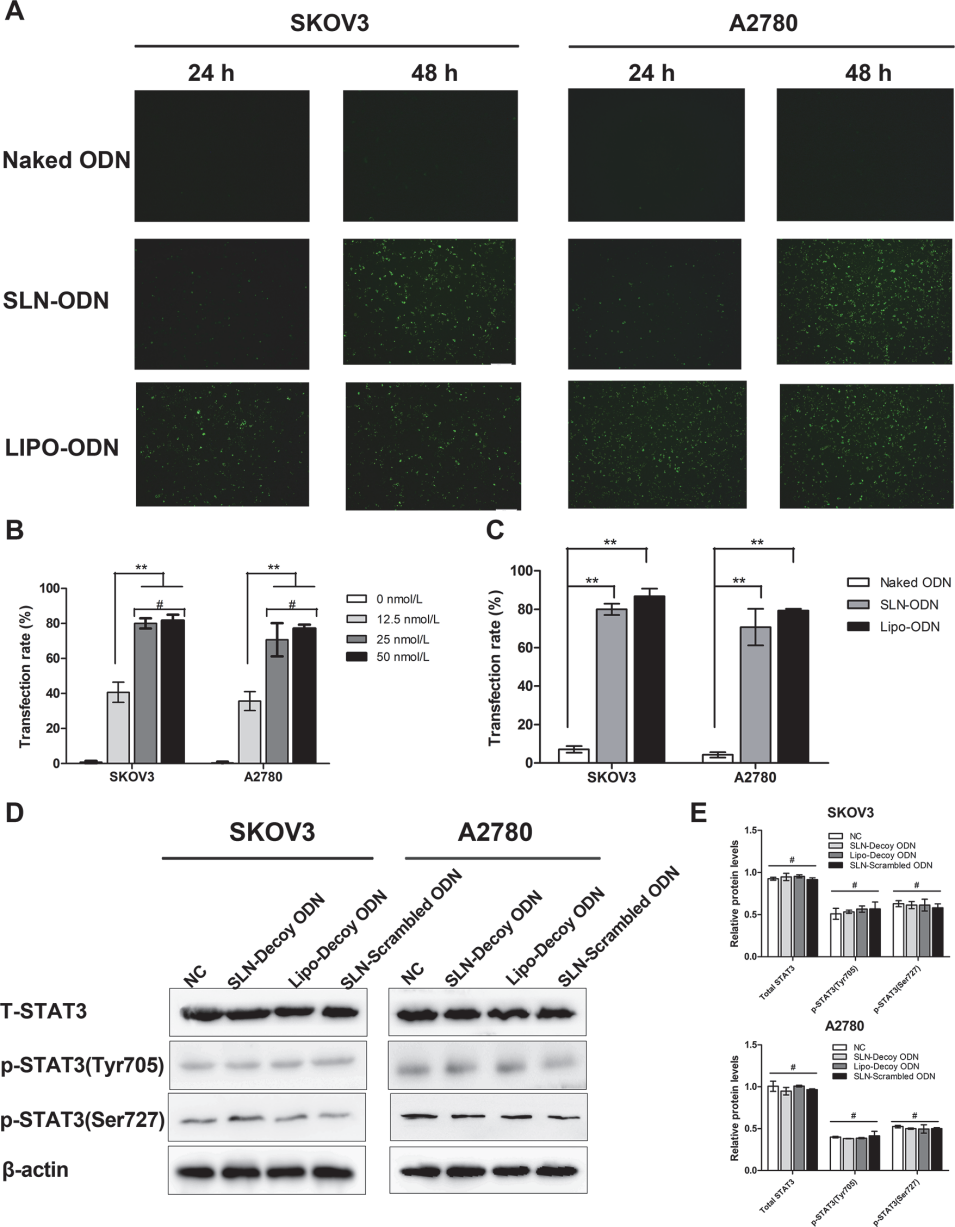
Uptake of SLN-decoy ODN complexes in ovarian cancer cells. (A) Fluorescent micrographs of SKOV3 and A2780 cells transfected with naked decoy ODN, SLN-decoy ODN and Lipofectamine 2000-decoy ODN at a concentration of 25 nmol/L at 24 and 48 hours post transfection. (B) Flow cytometry analysis of cellular uptake of SKOV3 and A2780 cells transfected with SLN-decoy ODN complexes at concentrations of 0, 12.5, 25 and 50 nmol/L at 48 hours post transfection. (C) Flow cytometry analysis of cellular uptake of SKOV3 and A2780 cells transfected with naked decoy ODN, SLN-decoy ODN and Lipofectamine 2000-decoy ODN complexes at a concentration of 25 nmol/L at 48 hours post transfection. (D) and (E) Western blot analysis of STAT3 and p-STAT3 (Ser727, Tyr705) protein expression. The relative protein levels were expressed as the ratio of protein of interest/β-actin. The data are expressed as the means ± SD of three independent experiments, ** *P* < 0.01, # *P* > 0.05.

Under a fluorescence microscope, the green fluorescence of the FITC-labeled decoy ODN marker was visualized. Both the SLN-decoy ODN complexes and the Lipo-decoy ODN complexes were successfully taken up by SKOV3 and A2780 cells (Fig 3A). A high cellular uptake of SLN-ODN complexes was obtained 48 hours after treatment. The cellular uptake of SLN-decoy ODN complexes was lower than that of Lipo-decoy ODN complexes at 24 hours but was similar at 48 hours after treatment. Flow cytometry was used to further quantify the uptake amount of SLN-decoy ODN after 48 hours of treatment (Fig 3B and 3C). The intracellular uptake of SLN-decoy ODN complexes exhibited a dose-dependent pattern; the cellular uptake was higher at concentrations of 25 and 50 nmol/L than that at 12.5 nmol/L (*P* < 0.01). However, no significant difference was observed between the 25 nmol/L and 50 nmol/L groups (*P* > 0.05). We chose 25 nmol/L for the following experiments. At this concentration, compared with that observed for naked ODN, higher cellular uptake was detected in SLN-decoy ODN (SKOV3 80%, A2780 70.73%) and Lipo-decoy ODN (SKOV3 86.7%, A2780 79.33%) complexes (*P* < 0.01). The cellular uptake of SLN-decoy ODN complexes was comparable to that of Lipo-decoy ODN (*P* > 0.05). This result indicated that the SLN-decoy ODN complexes could be effectively taken up by SKOV3 and A2780 cells. Western blot assays showed no differences in the expression of STAT3 and p-STAT3 among the four groups (Fig 3D and 3E). This result demonstrated that the mechanism of the complexes did not affect the expression levels of STAT3 and p-STAT3 but did affect the downstream target genes.

### SLN-STAT3 decoy ODN complexes induce apoptosis of ovarian cancer cells

**Fig 4:**
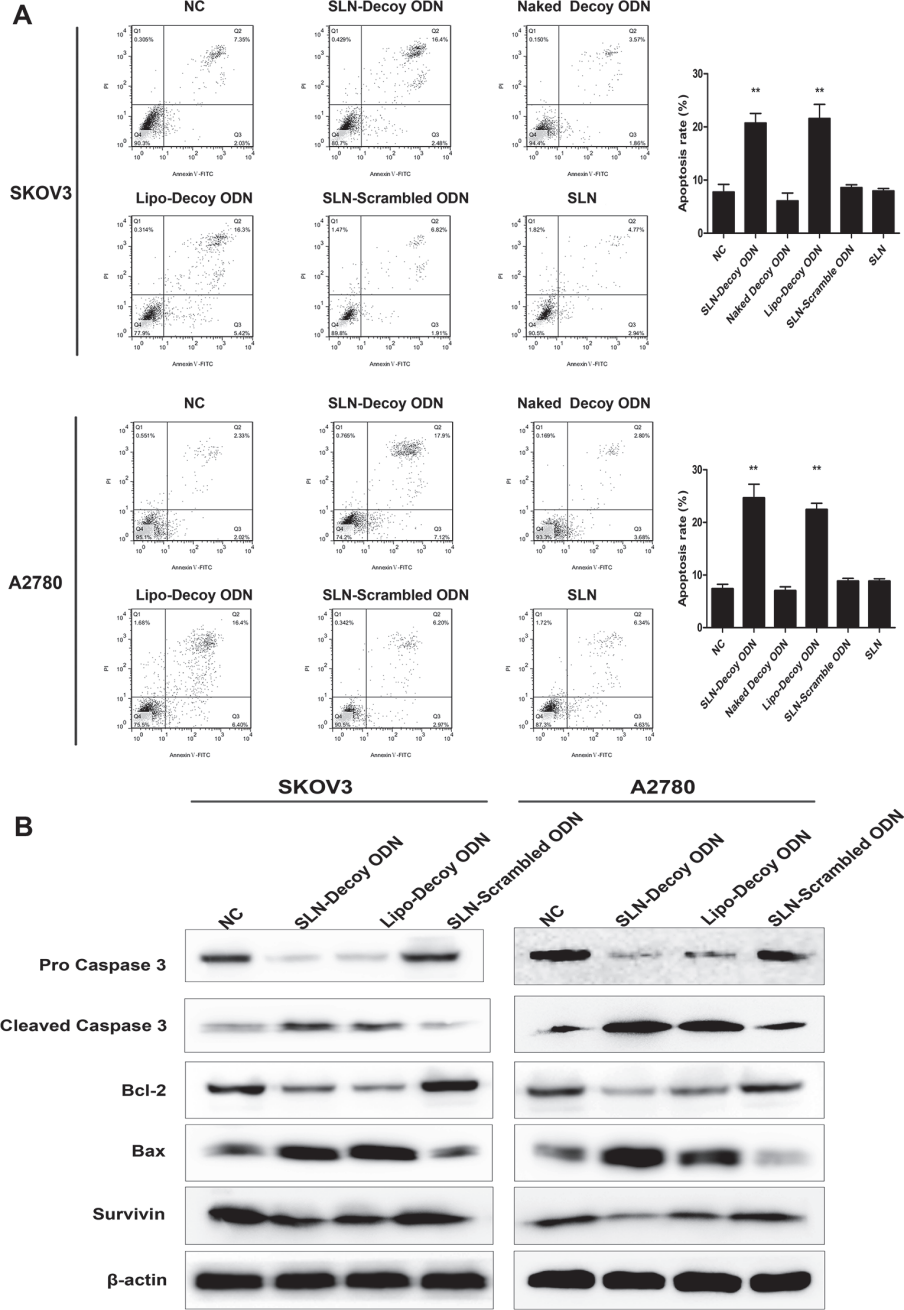
SLN-STAT3 decoy ODN complexes induce apoptosis of ovarian cancer cells. (A) SLN-decoy ODN induced a significant increase in apoptosis in SKOV3 and A2780 cells. After 48 hours of transfection, cell apoptosis was detected by annexin V-FITC/PI double-staining assay with flow cytometry. (B) Western blot assays showed that SLN-decoy ODN led to distinct up-regulation of Bax and cleaved caspase 3 and down-regulation of Bcl-2, pro-caspase 3 and Survivin. The data are expressed as the means ± SD of three independent experiments, ** *P* < 0.01 compared with NC.

To investigate the effect of the SLN-decoy ODN on cell apoptosis, the apoptotic rate was quantified through annexin V-FITC and PI double staining. Compared with those of the control groups, the apoptotic rates of the SLN-decoy ODN complexes (SKOV3 20.74%; A2780 24.64%) and Lipo-decoy ODN (SKOV3 21.59%; A2780 22.41%) were notably higher (*P* < 0.01; Fig 4A). These findings indicated that the SLN-decoy ODN complexes could affect the apoptotic program. The data also suggested that the apoptosis induction effect of SLN-decoy ODN was not due to the nanoparticle carrier because the SLN did not increase the apoptotic rate. To further explore the potential molecular mechanisms by which the SLN-decoy ODN complexes induce cell apoptosis, apoptosis-related proteins were detected by Western blot assays (Fig 4B). The results showed that the expression of Bax and cleaved caspase 3 were significantly increased in the SLN-decoy ODN and Lipo-decoy ODN groups. Furthermore, the protein levels of Bcl-2, pro-caspase 3 and Survivin were markedly reduced in the two abovementioned groups in both SKOV3 and A2780 cells.

### SLN-STAT3 decoy ODN complexes induce autophagy of ovarian cancer cells

**Fig 5:**
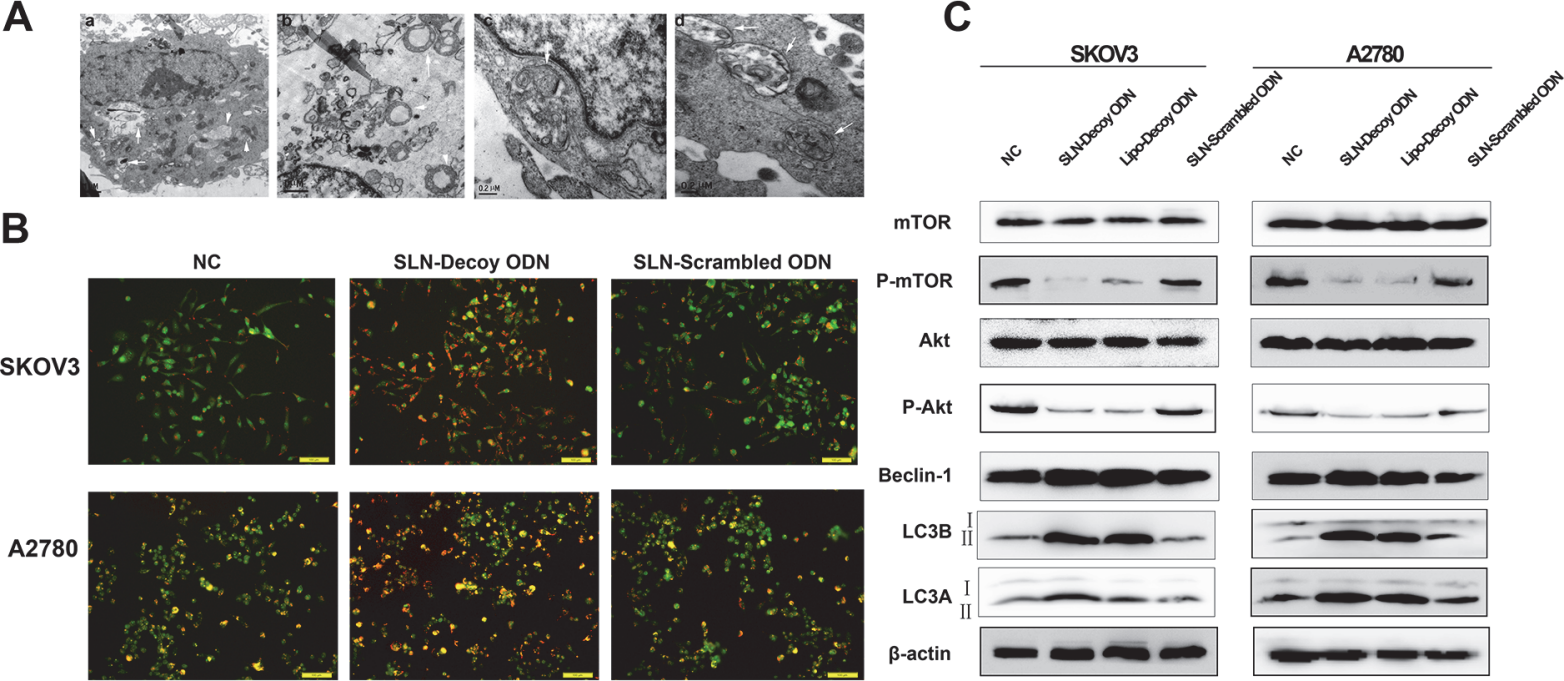
SLN-STAT3 decoy ODN complexes induce autophagy of ovarian cancer cells. (A) Transmission electron micrographs showing autophagic vesicles (AVs; white arrows) after transfection with the SLN-STAT3 decoy ODN in A2780 (a) and SKOV3 (b) cells. (c) Double-membrane AVs. (d) AVs containing cytosolic components. (B) Acridine orange staining at 48 hours after treatment in each group. (D) Western blot analysis showed that SLN-STAT3 decoy ODN reduced the expression of p-Akt, p-mTOR and increased the expression of LC3A-II, LC3B-II and Beclin-1.

TEM images revealed a large number of autophagic vesicles (AVs) in the cytoplasm of A2780 cells (Fig 5Aa) and SKOV3 cells (Fig 5Ab) at 48 hours after transfection of SLN-decoy ODN complexes. Under high magnification, double-membrane AVs were observed (Fig 5Ac), and AVs containing cytosolic components were found (Fig 5Ad). After acridine orange staining (Fig 5B), SLN-decoy ODN complexes showed considerable red fluorescence, while the other control groups displayed predominantly green fluorescence with very little red fluorescence. This result indicated an increased number of AVs as a consequence of an increase in autophagy. Furthermore, we explored autophagy on the molecular level (Fig 5C); the expression of LC3A-II and that of LC3B-II which are autophagosome-specific markers, increased after being transfected with SLN-decoy ODN and Lipo-decoy ODN. This result indicated an increased level of autophagy. To understand the mechanism of SLN-decoy ODN complexes induced autophagy, we observed that SLN-decoy ODN complexes resulted in a noticeable decrease in the expression of p-Akt and p-mTOR, whereas Beclin-1 expression increased. The protein level of total Akt was not decreased. The expression of total mTOR was slightly decreased in SLN-decoy ODN complexes group in SKOV3 cells, but the change was not significant in the four groups (*P* > 0.05). This result demonstrated that the SLN-decoy ODN complexes play a role in autophagy via regulating Beclin-1 expression and through the Akt/mTOR pathway.

### SLN-STAT3 decoy ODN complexes inhibit ovarian cancer cell migration and invasion

**Fig 6:**
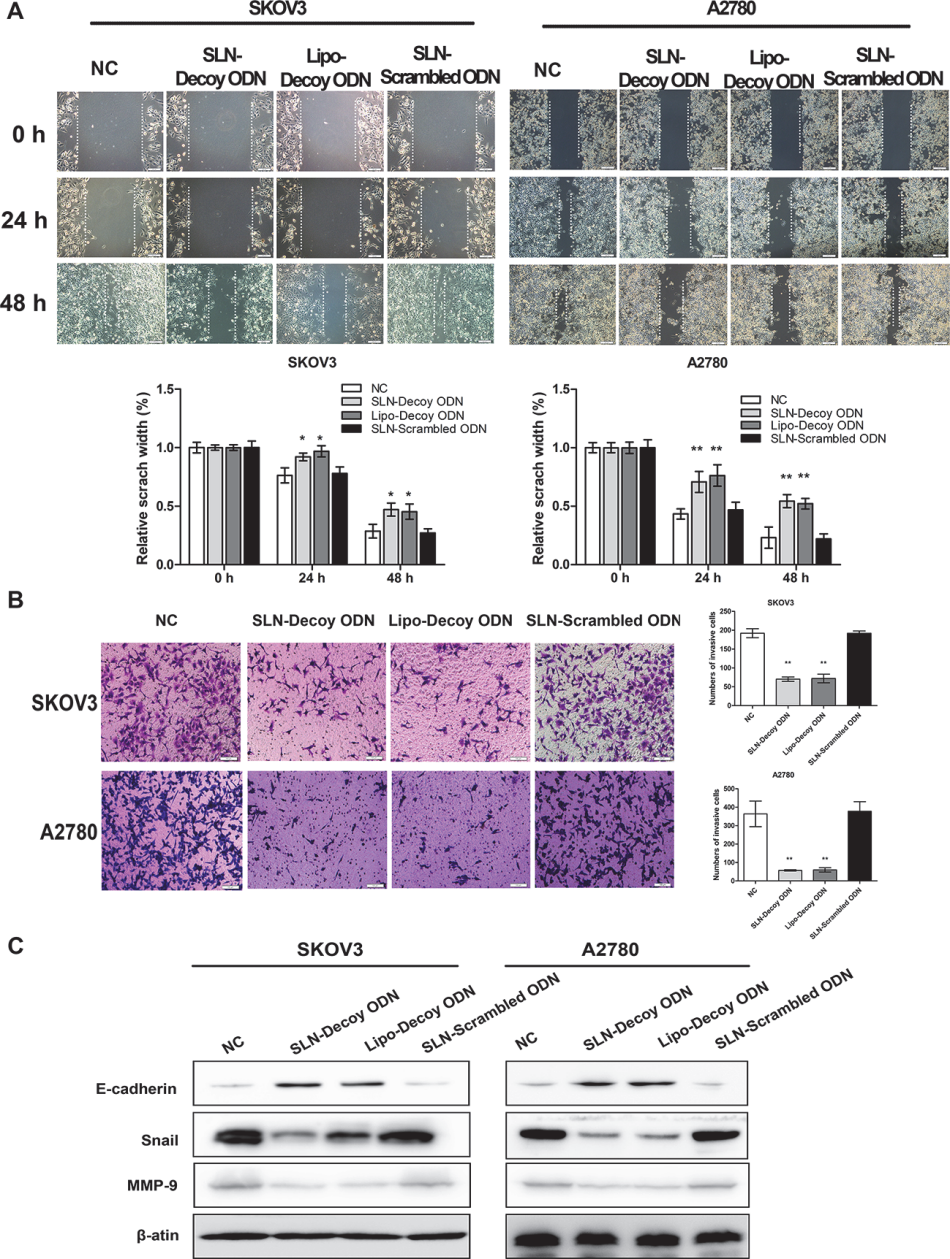
SLN-STAT3 decoy ODN complexes inhibit migration and invasion of ovarian cancer cells. (A) SLN-decoy ODN exhibited slower wound recovery at 24 and 48 hours after wounding compared with the NC and scrambled ODN groups. (B) A transwell invasion assay showed that SLN-decoy ODN inhibited cell invasion. At 48 hours after transfection, cells were reseeded to the upper transwell chamber for 24 hours and stained with crystal violet. (C) Western blot analysis showed that SLN-decoy ODN increased E-cadherin expression and reduced Snail and MMP9 expression. The data are expressed as the means ± SD of three independent experiments, * *P* < 0.05, ** *P* < 0.01 compared with NC.

A wound-healing assay showed, 24 and 48 hours after scratching, that the SLN-decoy ODN- and the Lipo-decoy ODN-transfected groups displayed a reduced wound closure compared with the control groups (SKOV3 *P* < 0.05; A2780 *P* < 0.01; Fig 6A). A transwell assay showed that the number of cells that passed through the membrane in the two abovementioned groups was significantly less than that in the two control groups (*P* < 0.01; Fig 6B). These findings indicate that the migration and invasion of the SLN-decoy ODN- and Lipo-decoy ODN-treated tumor cells were attenuated, whereas the other groups did not exhibit this attenuation. To further explore the molecular mechanisms by which SLN-decoy ODN complexes inhibit cell invasion, Western blot assays were performed. In the two abovementioned groups, the expression of Snail and MMP9 was down-regulated, and E-cadherin expression was up-regulated (Fig 6C). This result indicated that the SLN-STAT3 decoy ODN complexes could inhibit cell invasion via STAT3/Snail signaling and down-regulation of MMP9.

## Discussion

Understanding of STAT3 functions is helpful in identifying a potential target for tumor treatment. Our group has been interested in the use of STAT3 decoy ODN as a therapeutic approach to block the STAT3 pathway by binding to phosphorylated STAT3 and down-regulating STAT3 downstream oncogenes. The decoy ODN is a novel tool that has the advantages of specificity, effectiveness, stable sequence and low cost [28]. However, the essence of STAT3 decoy ODN is an oligonucleotide sequence that is nuclease-labile and exhibits poor cellular uptake. Carriers for ODN are necessary to overcome these issues.

An effective and safe delivery system for nucleic acids must possess the two following necessary characteristics [29]: 1) protection of encapsulated nucleic acid molecules from degradation by nucleases; and 2) possession of a cationic component that facilitates contact with anionic nucleic acid molecules. Additional useful properties include outstanding cellular uptake and drug accumulation at the target tumor site. To date, the most common systems for combining nucleic acid molecules for gene delivery involve viral vectors, liposomes or polymers, all of which exhibit high cellular uptake efficiencies. However, certain drawbacks impede further application due to the potentially toxic materials used, poor stability or the immune response caused by viral vectors. This research is far from complete. Moreover, even if polymer-based nanoparticles and cationic liposomes and SLN are promising delivery systems with high cellular uptake efficiency, the combination of decoy ODN-based therapeutics with these carriers has not been thoroughly investigated. In the present study, SLN loaded with decoy ODN were verified, for the first time, to exhibit enhanced delivery efficiency over their naked forms. It has been reported that the cellular uptake of microRNA-loaded SLN is slightly lower than that of Lipo-microRNA [29]. However, in our work, the cellular uptake efficiency of SLN-decoy ODN was comparable to that of Lipo-decoy ODN (P > 0.05). This result confirms the high delivery efficiency of SLN-decoy ODN.

SLN consist of natural lipid materials, and the nanodisperse system contains a solid matrix of crystalline solid lipids, capable of being highly taken up by cells and protecting nucleic acid molecules from degradation by nucleases [30, 31]. In the present study, CTAB, which is used as a cationic surfactant, was incorporated in to the particle surface at the time of emulsification and provided a highly positive zeta potential [32]. CTAB could facilitate gene loading and attachment between the nanoparticles and the cell surface. SLN have many advantages over other nanoformulations, such as easier preparation, safe component materials, low cost, better stability and controlled drug release [33, 34].

The toxicity of SLN is a crucial concern in biological applications. It has been reported that general uncharged SLN have no cytotoxic effects *in vitro* when the lipid concentration reaches 2.5% [35, 36]. The cytotoxicity of SLN is primarily due to the cationic surfactants, emulsifiers and preservatives that are used in the preparation of nanoparticles [37, 38]. In our work, the SLN prepared were practically non-toxic (Fig 2).

Apoptosis is one form of programed cell death that plays a pivotal role in cancer therapy. We detected significantly elevated expression of Bax and cleaved Caspase 3 but reduced expression of Bcl-2, pro-caspase 3 and Survivin in the SLN-STAT3 decoy ODN group (Fig 4). Bax and Bcl-2 participate in pro-apoptotic and anti-apoptotic signaling, respectively, and are pivotal regulators of apoptosis [39]. Caspase-3 is a central effector caspase, and cleaved caspase 3 participates in nuclear changes during apoptosis [40]. Survivin inhibits apoptosis by inhibiting caspases [41]. These data suggest that the SLN-STAT3 decoy ODN complexes induce apoptosis through intrinsic pathways.

Autophagy is a programmed cell survival process that can be regulated by the mTOR pathway [42], autophagy genes (ATG) and Beclin-1 [43]. STAT3 has been identified as a new autophagy regulator [44]. We confirmed the correlation of STAT3 and p-Akt. The down-regulation of p-Akt and p-mTOR led to the inhibition of the Akt/mTOR pathway. The lack of Akt/mTOR resulted in the transcriptional activation of ATG genes and mediated phosphorylation of proteins necessary for autophagosome formation. In parallel, we observed an up-regulation of Beclin-1, which is recognized as representation of autophagy [45].

Numerous recent studies have revealed that the epithelial to mesenchymal transition (EMT) plays an important role in tumor cell invasion and metastasis [46]. E-cadherin is an epithelial marker, and this calcium-dependent cell adhesion molecule is associated with the formation of adhesion connections [47]. The down-regulation of E-cadherin is characteristic of the EMT process [48]. Snail is a zinc-finger transcription regulator that inhibits the transcription of E-cadherin and initiates EMT [49]. Our results indicate that the SLN-STAT3 decoy ODN complexes inhibited the EMT program through STAT3/Snail signaling. We also observed down-regulation of MMP-9, which is considered crucial for cancer cell invasion.

In summary, we have successfully prepared a solid lipid nanoparticle system featuring STAT3 decoy ODN for ovarian cancer therapy. SLN-STAT3 decoy ODN complexes protect the integrity of the decoy ODN from degradation and increase the cellular uptake efficiency *in vitro*. Consequently, the complexes result in growth suppression and induction of apoptotic and autophagic cell death. Moreover, we observed that SLN-STAT3 decoy ODN complexes inhibit cell invasion. Taken together, the results indicate that the blockage of the STAT3 pathway by STAT3 decoy ODN carried by SLN may be a novel strategy for ovarian cancer treatment. Future studies will address the surface-modification of SLN to reduce cytotoxicity and achieve active targeting. In addition, the effects and mechanisms of SLN-STAT3 decoy ODN complexes *in vivo* will be further investigated.
